# Separation of ^103^Ru from a proton irradiated thorium matrix: A potential source of Auger therapy radionuclide ^103m^Rh

**DOI:** 10.1371/journal.pone.0190308

**Published:** 2017-12-22

**Authors:** Tara Mastren, Valery Radchenko, Philip D. Hopkins, Jonathan W. Engle, John W. Weidner, Roy Copping, Mark Brugh, F. Meiring Nortier, Eva R. Birnbaum, Kevin D. John, Michael Ernst-Heinrich Fassbender

**Affiliations:** 1 Chemistry Division, Los Alamos National Laboratory, Los Alamos, New Mexico, United States of America; 2 Nuclear Security and Isotope Technology Division, Oak Ridge National Laboratory, Oak Ridge, Tennessee, United States of America; University of Liverpool, UNITED KINGDOM

## Abstract

Ruthenium-103 is the parent isotope of ^103m^Rh (t_1/2_ 56.1 min), an isotope of interest for Auger electron therapy. During the proton irradiation of thorium targets, large amounts of ^103^Ru are generated through proton induced fission. The development of a two part chemical separation process to isolate ^103^Ru in high yield and purity from a proton irradiated thorium matrix on an analytical scale is described herein. The first part employed an anion exchange column to remove cationic actinide/lanthanide impurities along with the majority of the transition metal fission products. Secondly, an extraction chromatographic column utilizing diglycolamide functional groups was used to decontaminate ^103^Ru from the remaining impurities. This method resulted in a final radiochemical yield of 83 ± 5% of ^103^Ru with a purity of 99.9%. Additionally, measured nuclear reaction cross sections for the formation of ^103^Ru and ^106^Ru via the ^232^Th(p,f)^103,106^Ru reactions are reported within.

## Introduction

Rhodium-103m (t_1/2_ 56.12 m) is an isotope of interest for targeted radiotherapy that decays via isomeric transition to stable ^103^Rh. This decay gives rise to the emission of low-energy Auger/Coster-Kronig electrons (2.3 electrons/decay) [1], which have the potential to induce double stranded DNA damage promoting cancer cell death. Rh-103m was identified by Bernhardt et al. as one of five radionuclides of interest for auger therapy that would deliver a sufficiently high dose to the tumor with minimal dose to surrounding tissue as the energy of the emitted electrons are lower than 40 keV and the half-life is sufficient for targeting [2].

The use of Auger/Coster-Kronig electrons for targeted radiotherapy is an emerging field of nuclear medicine [3, 4]. The linear energy transfer (LET) in biological tissue by these low-energy electrons is high due to the short path length (nm scale). High LET, i.e. energy release, provided within a short range is ideal for targeted radiotherapy, which potentially kills the cancer or diseased cells in the vicinity of the decay and provides less damage to surrounding healthy tissue. Due to the short ranges of auger electrons it is important that the targeting agent is internalized by the cell to where it can do the most damage [5].

The pathway to the production of ^103m^Rh leads via formation of its parent radionuclides ^103^Pd (t_1/2_ 16.99 d) and ^103^Ru (t_1/2_ 39.21 d). Production of the parent isotopes permit the use of a generator system to supply ^103m^Rh [6–9]. Production of ^103^Pd using proton or deuteron induced reactions on ^103g^Rh (stable) via ^103g^Rh(p,n)^103^Pd and ^103g^Rh(d,2n)^103^Pd has been investigated [10–12]. Other production routes include the neutron irradiation of ^102^Ru (stable) to produce ^103^Ru as well as the isolation of ^103^Ru from fission products of ^235^U through the following reactions: ^102^Ru(n,γ)^103^Ru and ^235^U(n,f)^103^Ru [6, 7].

Currently, Los Alamos National Laboratory (LANL), Oak Ridge National Laboratory (ORNL), and Brookhaven National Laboratory (BNL) are investigating the bulk production of ^225^Ac (t_1/2_ 9.92 d), another isotope of interest for therapeutic applications, by proton irradiations of thorium targets [13, 14]. During this process many additional isotopes of interest are generated, including fission products like ^103^Ru. Methods are being investigated to leverage these irradiations to recover additional radionuclides of interest to nuclear medicine without a negative impact on the ^225^Ac recovery process.

The simultaneous separation of ^103^Ru and ^225^Ac from bulk thorium and fission products has three main challenges: 1) the separation of ^103^Ru from a mixture containing bulk thorium and many additional fission products, 2) minimization of impacts to the ^225^Ac purification process, and 3) management of the multiple oxidation states of ^103^Ru. In this work, we introduce an anion exchange method that has been developed to address these challenges. It has been successfully used to isolate ^103^Ru in high yield.

Nuclear excitation functions, i.e., nuclear reaction cross sections as a function of proton energy, reflect the probability at which a desired nuclear reaction occurs. Carefully measured excitation functions provide the radionuclide production scientist with an invaluable tool to estimate both product yield and expected levels of unwanted byproducts. Target thicknesses, particle energies and separation chemistry design are developed according to excitation function based estimates. Hence, to provide a link between the analytical data of this work and the application to radionuclide production science, the excitation functions for the proton induced formation of ^103^Ru and ^106^Ru through the reactions ^232^Th(p,f)^103^Ru and ^232^Th(p,f)^106^Ru, respectively, are reported as well at incident energies below 200 MeV. Excitation functions were calculated by the analysis of data collected from previous thin foil activation experiments [15] conducted at the Los Alamos Neutron Science Center’s 100 MeV and 200 MeV proton beams. These energy differential cross sections were used to obtain predicted thick target yields of up to 111 GBq (3 Ci) for 100 g thorium targets (thickness 8.6 mm) after exposure to 49.9 mAh of integrated proton beam current, the projected fluence to which thorium targets will be exposed for ^225^Ac production at the Isotope Production Facility (IPF) at LANL.

## Materials and methods

### Materials

All reagents used were trace metal grade unless specified elsewhere. Aqueous solutions were prepared with 18 MΩ water (Millipore) on site. AG1-X8 resin was obtained from Biorad (Hercules, CA, USA) and DGA resin (N,N,N′,N′-tetra-n-octyldiglycolamide) was obtained from Eichrom (Lisle, IL, USA). For nuclear reaction cross section measurements, natural thorium foils of 99.7% purity were obtained from Goodfellow Corporation (Oakdale, PA, USA). The foils were approximately 2.5 × 2.5 mm, with thicknesses of 60.5–70.5 mg/cm^2^.[15] Aluminum foils of 99.9% purity and similar dimensions with 65 mg/cm^2^ thickness were added to the target foil stack as beam monitors using the ^27^Al(p,x)^22^Na nuclear reaction and the excitation functions reported by Steyn et al.[16] Foils were enclosed in a single layer of adhesive-backed 25 μm thick Kapton tape. Thorium metal targets were manufactured at Los Alamos National Laboratory (LANL). For larger-scale experiments, small pieces of thorium metal (purity >99% as determined via X-ray fluorescence spectroscopy) were obtained from LANL’s internal inventory. The raw material was arc melted and rolled into sheets with a mean thickness of 0.50 ± 0.02 mm for the use as proton beam targets. All separation studies reported within were performed in triplicate.

### High Purity Germanium (HPGe) detector analysis

#### Cross section measurements

Proton induced fission cross sections for the production of ^103^Ru (t_1/2_ 39.21 d) and ^106^Ru (1.017 a) were extracted from previous work utilizing nondestructive γ-ray spectroscopy the activity of each residual radionuclide of interest after several hours’ delay to allow short-lived radionuclides to decay [15, 17]. The thorium foils were counted on an ORTEC GEM10P4-70 detector with a relative efficiency of 10%, while the aluminum foils were counted on a Princeton Gamma-tech lithium-drifted germanium Ge(Li) detector with a relative efficiency of 13.7%. Both detectors were well shielded and calibrated using National Institute of Standards and Technology (NIST)-traceable gamma calibration sources. The thorium foils were counted more than 35 times over a period of several months, and the decay curves of all isotopes of interest were closely followed to ensure proper identification and to evaluate any possible interferences. Ruthenium-106 decay is not accompanied by a γ-ray emission. Hence the 622 keV gamma from its daughter ^106^Rh (29.8 s) was used for cross section calculations as ^106^Rh is in secular equilibrium with ^106^Ru. The aluminum foils were counted approximately 12 times within the first week after end of bombardment (EOB) to monitor the ^24^Na decay curve, followed by a minimum of three 8 h counts several weeks later to quantify the ^22^Na activity at EOB. Uncertainties in linear regressions fitted parameters were computed from covariance matrices as the standard deviation in the activity extrapolated to the end of bombardment. This value was combined according to the Gaussian law of error propagation with estimated contributing uncertainties from detector calibration and geometry reproducibility (5.9% combined), target foil dimensions (0.1%), and proton flux (6%–18%). Multiple photopeaks were used (up to a maximum of 4) when possible, and so additional uncertainty as the standard deviation of these complementary measurements was combined with the uncertainties described above, again according to the Gaussian law of error propagation.

#### Separation chemistry analysis

Gamma-ray spectroscopy chemical separation experiments was conducted using an EG&G Ortec Model GMX-35200-S HPGe detector system in combination with a Canberra Model 35-Plus multichannel analyzer. Detector diameter was 50.0 mm, detector length was 53.5 mm, Be window thickness was 0.5 mm, and outer dead-layer thickness was 0.3 μm. Detector response function determination and evaluation were performed using standards of radionuclide mixtures containing ^241^Am, ^109^Cd, ^57^Co, ^139^Ce, ^203^Hg, ^113^Sn, ^137^Cs, ^88^Y, and ^60^Co, traceable to the NIST and supplied by Eckert & Ziegler (Atlanta, GA, USA). The detector was a p-type Al-windowed HPGe detector with a measured FWHM at 1333 keV of approximately 2.2 keV and a relative efficiency of about 10%. Relative total source activity uncertainties ranged from 2.6% to 3.3%. Counting dead times were kept below 10%.

### Cross section measurement irradiations

Thin thorium foils were irradiated in two separate experiments at the Los Alamos Neutron Science Center (LANSCE) at LANL using incident proton energies of 100 and 200 MeV as described previously [15]. In each experiment, the original beam energy was degraded to approximately half of its original value using aluminum degraders. Beam current was monitored using thin aluminum foils and evaluated cross sections for the ^27^Al(p,x)^22^Na reaction [https://www-nds.iaea.org/exfor/servlet/X4sSearch5]. The beam profile was assessed following the experiment by the activation of thin stainless steel plates whose dimensions significantly exceeded those used for the thorium foils. The steel plates were exposed to Gafchromic^®^ film following the end of irradiation in order to map the beam profile, which was determined to have been quantitatively incident on the desired targets in both experiments.

### Production target irradiations

A 10 g thorium metal target was irradiated at the Isotope Production Facility (IPF), Los Alamos National Laboratory (LANL, NM, USA). The target was encapsulated in Inconel cladding and placed into the high energy “A” slot (nominal 92 MeV incident energy) of the IPF target assembly. IPF targetry and 4π water cooling were identical to the design as described previously [18, 19]. The target was irradiated with 230 μA of 89.6 MeV protons for 22.5 hours.

### Separation of ^103^Ru

The irradiated 10 g thorium target was shipped to Oak Ridge National Laboratory (ORNL) for recovery of ^225^Ac. The target was dissolved in 200 mL 10 M HCl and 0.1 mL of 2M HF with heating (80–90°C) for approximately 2 hours. A 0.1 mL aliquot of the dissolved target was diluted to 5.1 mL with 0.1M HNO_3_. This solution was then used as a stock solution for radiotracers that represent radionuclides previously identified in the target [13]. For the chemical separation studies, a spiked mock-up solution was prepared. Approximately 1 g of thorium metal was dissolved with 20 mL 10 M HCl spiked with 40 μL 2 M HF. A 50 μL aliquot of the radiotracer stock solution, as prepared above, was then added to this solution and contacted with 10 g AG1-X8 resin in a plastic column (Biorad). The eluent was collected (fraction 1) and the column was washed with an additional 2 x 5 mL of 10 M HCl (fraction 2 &3). The column was then washed with four 5 mL fractions of 1 M HCl (fractions 4–7). To elute the remaining ^103^Ru, eight 5 mL fractions of 10 M HNO_3_ were added to the column and collected (fractions 8–15). All fractions were analyzed by HPGe spectroscopy using the characteristic γ-rays as identified in Table 1. Fractions 1–3 were brought to soft dryness and reconstituted in 205 mL 1 M citric acid solution, adjusted to pH 2 with HCl, and subjected to the ^225^Ac purification process as described previously [13, 14].

**Table 1:** Fission product nuclides identified in this study.

Radionuclide	Half-life (d)	Identifying γ-ray Emissions (keV) [% Intensity]
^103^Ru	39.27	497 [90.9]
^95^Nb	34.98	765 [100]
^95^Zr	64.02	724 [44.17] / 756 [54]
^117m^Sn	13.6	158.56 [86]
^123m^Te	119.7	158.97 [84]
^121^Te	154	212 [81]
^233^Pa	26.97	312 [38.6]
^124^Sb	60.2	602.7 [98.3] / 1690 [47.8]

A second column was developed to remove contaminants from the ^103^Ru eluted in fractions 8–15. Contaminants present included ^95^Zr, ^95^Nb, ^233^Pa, ^230^Pa, ^117m^Sn and ^124,125^Sb. These fractions were brought to near dryness and reconstituted in 10 mL 10 M HCl. This solution was then passed through a column containing 1 mL DGA (N,N,N′,N′-tetra-n-octyldiglycolamide) equilibrated with 10 M HCl. The eluent was collected (fraction 16) and the column was washed with an additional 20 mL 10 M HCl (fraction 17). All separation experiments were performed in triplicate.

## Results and discussion

### Cross section measurements

**Fig 1:**
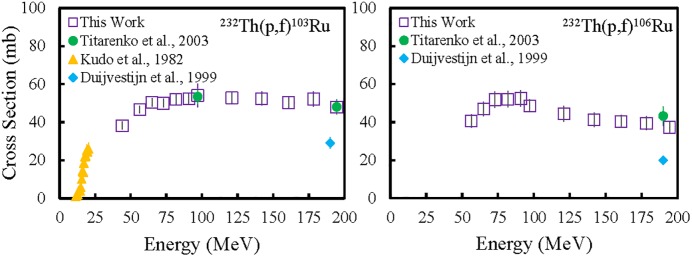
Measured excitation functions for the formation of ^103^Ru (left) and ^106^Ru (right) for proton energies less than 200 MeV [20–22].

Measured excitation functions of ^103^Ru, and ^106^Ru are plotted in Fig 1 along with literature data [20–22]. The cross sections obtained in this work and the corresponding uncertainties are listed in Table 2. The cross sections reported in this work are similar to those measured by Titarenko et al. [21] and Kudo et al. [20] and slightly higher than those measured by Duijvestijn et al. [22]. In addition to proton induced fission, neutron induced fission does occur and may have an impact on the data. The effect of neutron induced fission is understood to be small, as the secondary neutron fluence is smaller than that of the primary beam by several orders of magnitude. Because secondary neutrons’ angular distribution is forward-directed, their effect increases towards the “rear”, or lower energy, portion of a target foil stack. Details of these measurements have been extensively discussed previously [15, 17, 23, 24].

**Table 2:** Measured excitation functions for the ^232^Th(p,f)^103^Ru and ^232^Th(p,f)^106^Ru reactions.

Nominal energy (MeV)	^232^Th(p,f)^103^Ru (mb)	Uncertainty (mb)	^232^Th(p,f)^106^Ru (mb)	Uncertainty (mb)
194.5	48	4	37	3
178.3	52	4	39	4
160.7	50	3	40	3
141.8	52	4	41	4
120.9	53	3	44	4
97	54	6	48	3
90.8	52	3	52	4
81.7	52	3	52	4
72.8	50	2	52	4
64.9	50	2	47	4
56.3	47	2	41	3
44	38	2		

### Separation of ^103^Ru

**Fig 2:**
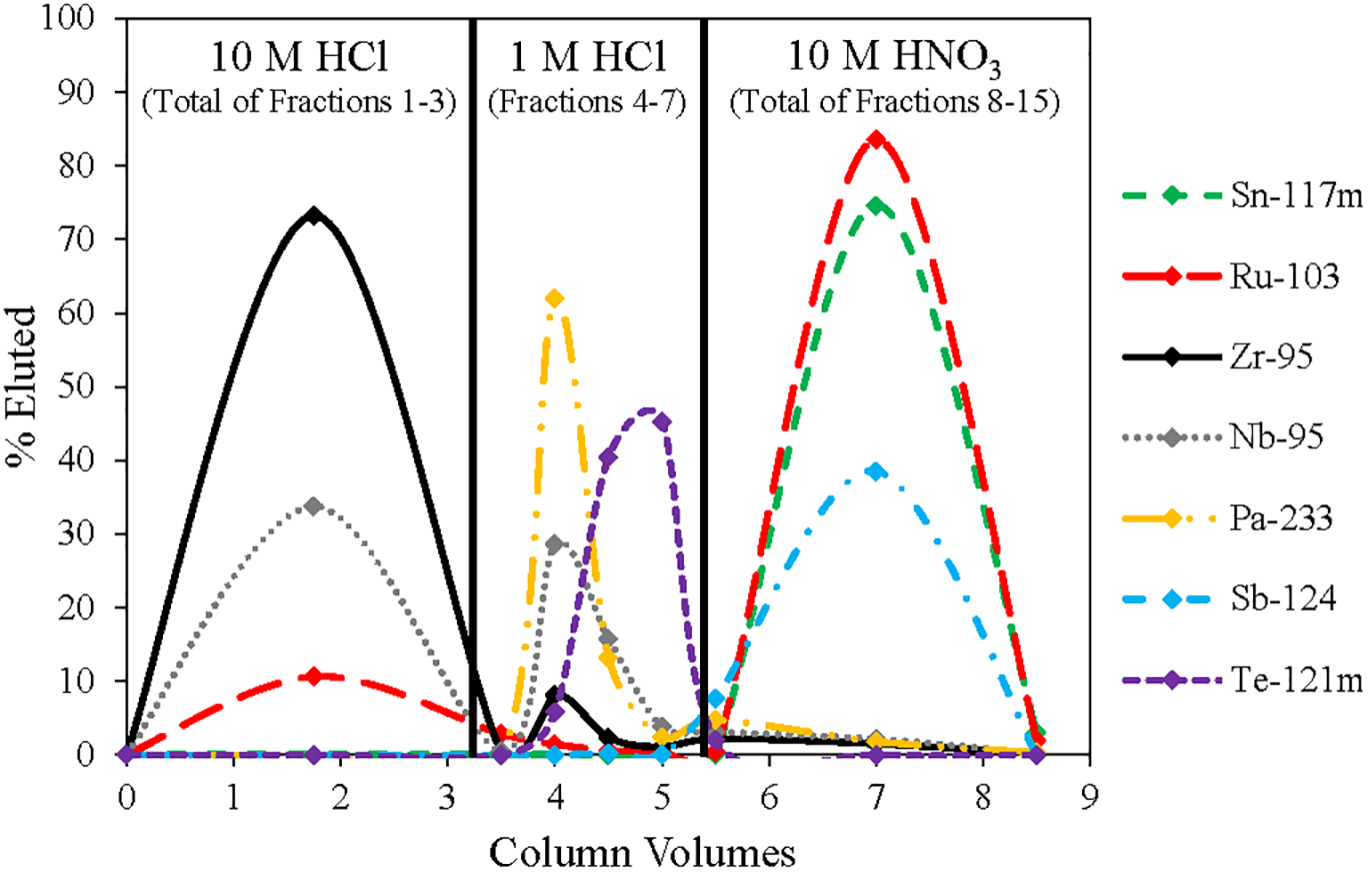
Elution profile of Sn, Pa, Ru, Sb, Zr, Nb, and Te on the anion exchange column. Fractions 1–3 are combined into one 10 M HCl fraction and fractions 8–15 are combined into one 10 M HNO_3_ fraction.

The cationic species such as ^225^Ac, Ra, Ba, lanthanides and bulk thorium along with the majority of ^95^Zr passed through the anion column in the loading fraction and 10 mL 10 M HCl wash (fractions 1–3). Ruthenium is most strongly retained on the anion exchange resin using 1 M HCl [25], while several other fission products are not retained. Therefore the column was washed with an additional 20 mL of 1 M HCl (fractions 4–7), resulting in the removal of the majority of the ^95^Nb and ^123m^Te along with approximately 45% of the Pa. The loading and washing of the column resulted in ^103^Ru losses of 8–15%. Thirty milliliters of 10 M HNO_3_ resulted in the elution of 85 ± 5% of ^103^Ru with a radiochemical purity of 82%. The main impurities present in this fraction consisted of ^117m^Sn and ^125,126^Sb with trace amounts of ^230,233^Pa, ^95^Nb, and ^95^Zr. Fig 2 shows the elution of Nb, Zr, Te, Sn, Sb, Pa and Ru from the anion column.

**Fig 3:**
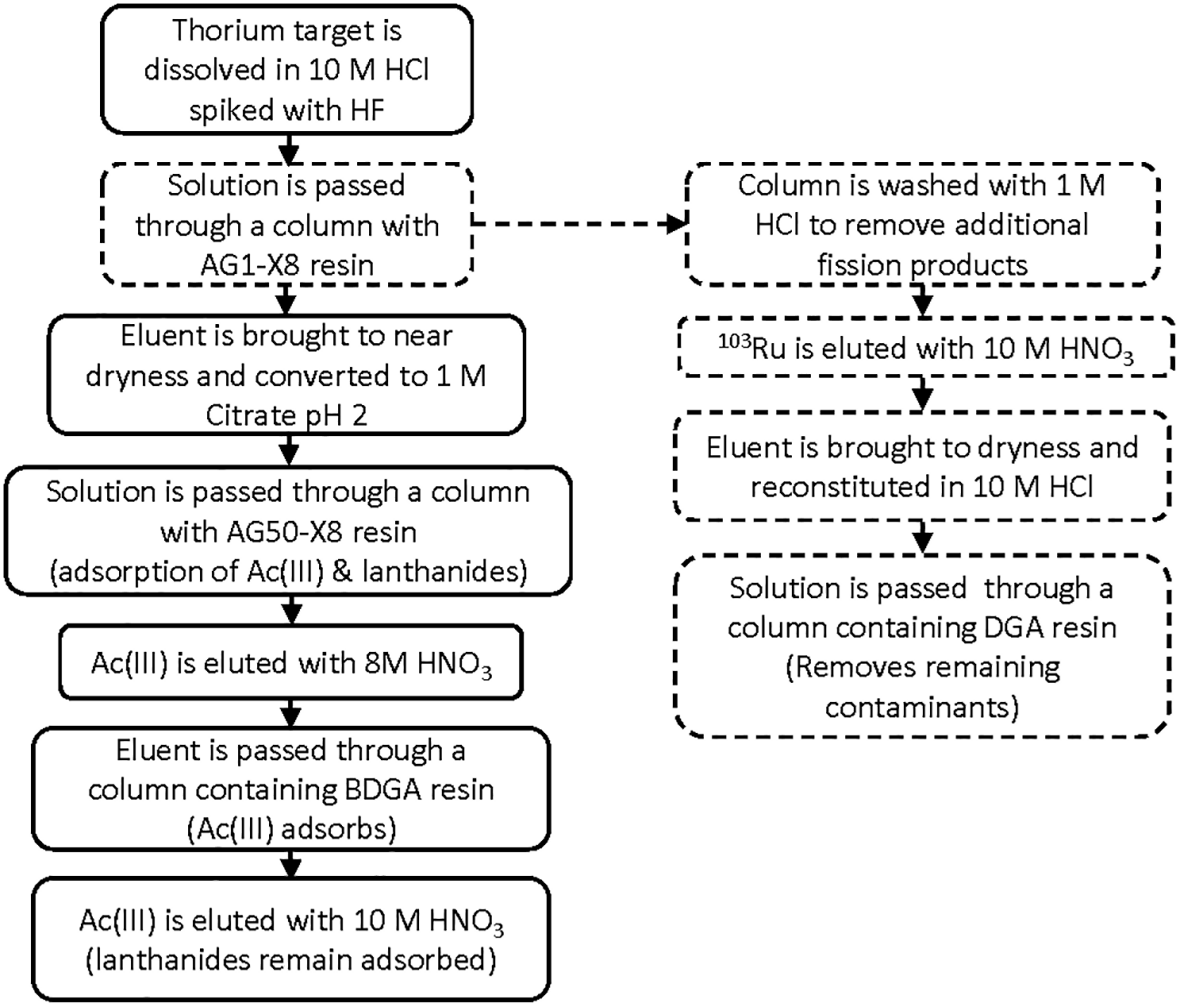
Separation schematic showing separation of ^103^Ru (dashed lines) in tandem with ^225^Ac separation (solid lines) [13,14].

According to a paper published by Pourmand et al. [26], Nb, Zr, Sn and Sb are strongly retained on DGA resin in high concentrations of HCl. Ruthenium, however is not strongly retained on DGA resin or in TODGA extraction systems with HCl or HNO_3_ solutions and several papers discuss fission product behavior in these systems [14, 27–32]. Therefore a DGA column was employed to remove these contaminants. The average recovery of ^103^Ru from the DGA column was 98 ± 1% resulting in a final ^103^Ru recovery of 83 ± 5% with a radiochemical purity of > 99.9%. Ruthenium speciation is a complicated subject with respect to its separation in acid based systems and is likely responsible for the high variability in the loss of ^103^Ru from the anion column (8–15%) [33]. Ideally, pretreatment of the solution to obtain one species of ruthenium would be advantageous for consistent recoveries, however pretreatment is not possible in this case as the isolation of ^103^Ru is an ancillary activity with respect to ^225^Ac recovery. A flow diagram of the whole process is shown in Fig 3.

As both ^117m^Sn and ^123m^Te have the same identifying γ-ray lines without a viable secondary gamma, a spike of ^121m^Te was added to the sample to help deconvolute the separation of ^123m^Te and ^117m^Sn. This information led to the determination that ^123m^Te was present in the 1 M HCl fractions and ^117m^Sn was present in the 10 M HNO_3_ fractions. This elemental distribution is further corroborated by prior reports that tellurium is eluted from anion columns in 1 M HCl while tin is retained strongly, and that tin elutes with ^103^Ru in 10 M HNO_3_ [34, 35].

Ruthenium-103 obtained from this method contains the isotopic impurity ^106^Ru. However as ^106^Ru decays to ^106g^Rh (29.9 s), ^103m^Rh obtained from a generator would be isotopically pure five minutes after elution. The predicted experimental yield calculated from measured cross sections of ^103^Ru, with anticipated full scale ^225^Ac production, is (~ 3 Ci (111 GBq)) at end of bombardment. This would significantly increase the current supply of ^103^Ru for medical research needs.

Future work needs to be performed in order to determine suitable conditions for a ^103^Ru/^103m^Rh generator system. Solvent extraction generators have been designed employing a carbon tetrachloride extraction, however given the toxicity associated with CCl_4_ this method is not amenable for biomedical applications [6, 36]. A successful generator would employ a solid support that allows repeated elution of ^103m^Rh with minimal breakthrough of ^103^Ru. This would preferably entail the use of mineral acids that can be readily removed from the product such as HCl or HNO_3_.

## Conclusions

A method was obtained for the recovery and purification of ^103^Ru that is produced concurrently with ^225^Ac. This method results in a final ^103^Ru chemical recovery yield of 83 ± 5% with a radiochemical purity of > 99.9%. The measurement of energy dependent cross sections for the proton induced fission production of ^103^Ru and ^106^Ru at proton energies less than 200 MeV on ^232^Th targets predict thick target yields of ~111 GBq. This process can be implemented with the existing ^225^Ac recovery flow sheet at minimal impact to the ^225^Ac process. Additionally, future work to develop a robust ^103^Ru/^103m^Rh radionuclide generator is essential to the success of ^103m^Rh for auger therapy.
